# Chemical Characteristics and Thermal Oxidative Stability of Novel Cold-Pressed Oil Blends: GC, LF NMR, and DSC Studies

**DOI:** 10.3390/foods12142660

**Published:** 2023-07-10

**Authors:** Wojciech Cichocki, Dominik Kmiecik, Hanna Maria Baranowska, Hanna Staroszczyk, Agata Sommer, Przemysław Łukasz Kowalczewski

**Affiliations:** 1 InnPlantFood Research Group, Poznań University of Life Sciences, 60-624 Poznań, Poland; 2 Department of Food Technology of Plant Origin, Poznań University of Life Sciences, 60-624 Poznań, Poland; 3 Department of Physics and Biophysics, Poznań University of Life Sciences, 60-637 Poznań, Poland; 4 Department of Chemistry, Technology and Biotechnology of Food, Chemical Faculty, Gdańsk University of Technology, 80-233 Gdańsk, Poland

**Keywords:** fatty acid composition, triacylglycerol polymerization, thermal stability, oil oxidation, molecular properties of oils

## Abstract

Plant oils contain a high content of unsaturated fatty acids. Studies of food products have revealed a considerable disproportion in the ratio of *ω*6 to *ω*3. This article presents information on the healthful qualities of eight new oil blends that contain a beneficial proportion of *ω*6 to *ω*3 fatty acids (5:1), as well as their degradation during heating at 170 and 200 °C. The fatty acid profile was analyzed by gas chromatography (GC), content of polar compounds and polymers of triacylglycerols by liquid chromatography (LC), water content was measured by the Karl Fischer method, and oxidative stability was measured by differential scanning calorimetry (DSC) and low-field nuclear magnetic resonance (LF NMR) methods. The results showed that during heating, the polar fraction content increased in samples heated at both analyzed temperatures compared to unheated oils. This was mainly due to the polymerization of triacylglycerols forming dimers. In some samples that were heated, particularly those heated to 200 °C, trimers were detected, however, even with the changes that were observed, the polar fraction content of the blends did not go beyond the limit. Despite the high content of unsaturated fatty acids, the analyzed blends of oils are characterized by high oxidative stability, confirmed by thermoanalytical and nuclear magnetic resonance methods. The high nutritional value as well as the oxidative stability of the developed oil blends allow them to be used in the production of food, in particular products that ensure an adequate supply of *ω*3 fatty acids.

## 1. Introduction

Plant-based oils and fats play a crucial role in maintaining good health. They supply energy, lipophilic vitamins such as A, D, E, and K, and other bioactive compounds. Plant-based fats are known for having a high amount of monounsaturated (MUFA) and polyunsaturated (PUFA) fatty acids [1]. The nutritional value (fatty acid profile, content of tocopherols, sterols, and phenolic compounds) and the quality of vegetable oils depend on many factors. The content of active compounds in the oil depends on both the cultivation conditions and the environment, but also one of the basic factors affecting the quality of the oil is the quality of the raw material subjected to pressing and the method of obtaining the oil [2,3]. Cold-pressed oils are obtained by hydraulic or screw presses at a temperature not exceeding 50 °C [4]. In the case of hydraulic presses, the pressed oil is exposed to greater contact with oxygen, which may cause quality deterioration due to auto-oxidation. During the use of screw presses, the seeds are subjected to increased temperature. The final stage is the purification of the oils, which can be carried out by sedimentation, centrifugation, or filtration [4,5]. Therefore, it is crucial to control the conditions of cultivation, storage of the raw material, and the oil extraction process.

The growing trend of consuming minimally processed foods has made cold-pressed oils increasingly popular, primarily due to their high nutritional value. An important parameter in the pressing process is maintaining a controlled temperature below 40 °C. This low temperature prevents loss MUFAs and PUFAs and also inhibits the extraction of unfavorable compounds from the seeds into the oil [6,7]. Due to the fact that cold-pressed oils are characterized by a high content of PUFAs, it should be remembered that the oxidation reaction significantly reduces the quality of the oil [8]. Oils that commonly present on the market may differ in the composition of PUFAs [9]. Often products consumed by humans may have an improper ratio of *ω*6/*ω*3, which, in consequence, may have an adverse effect on human health [10]. It should be remembered that too much consumption of *ω*6 fatty acids has a negative effect on the functioning of some organs and as a result causes disease, e.g., heart disease and other diseases. Omega-3 fatty acids, due to their involvement in prostaglandin metabolism, contribute to lowering cholesterol levels and also have anti-inflammatory and anti-coagulant properties. The global trend of vegan and vegetarian diets has made the correct ratio of *ω*6/*ω*3 acids even more important [11]. From a health point of view, optimal ratio of *ω*6/*ω*3 fatty acids is in the range 5:1 to 10:1 and may vary by geographic region [12]. A good solution is to mix oils and obtain blends with specific health properties. Food analyzes show that the ratio *ω*6/*ω*3 is often above 20:1 [11,13,14].

Vegetarians and vegans are particularly vulnerable to the deficiency of *ω*3 fatty acids in their diet. They do not eat fish and seafood, which are widely recognized as a good source of PUFAs, especially eicosapentaenoic (EPA, 20:5 cis-5,8,11,14,17) and docosahexaenoic (DHA; C22:6 cis-4,7,10,13,16,19) fatty acids [15]. Literature data, however, indicate that a well-planned plant-based diet can provide all the nutrients necessary for good health [16,17]. Therefore, it is particularly important to use oils of the right quality in the diet, which will ensure an adequate supply of essential unsaturated fatty acids.

Oxidation is a process that occurs in multiple stages and can be triggered by various factors such as oxygen, free radicals, heat, or light. Due to the oxidative processes that occur in food, the high content of unsaturated fatty acids can also be a problem, especially in the case of highly processed food or food with a long shelf life [18]. During food storage, the oxidation of fatty acids can lead to the production of volatile compounds that can significantly impact the deterioration of the food’s sensory properties [19,20]. Hence, the ability to inhibit oxidation is a crucial property of food. Antioxidants present in food help protect cells from the harmful effects of reactive oxygen species. Examples of antioxidant compounds in oils are carotenoids, polyphenols, phytosterols, and tocochromanols. Their strong antioxidant properties protect unsaturated fatty acids from oxidation [21,22].

The process of frying is one of the most popular methods of preservation and preparation food for consumption. Furthermore, the frying is quick, simple, and inexpensive. The factors that affect oil degradation are as follows: high temperature during frying, long use of one batch of oil, and also the type of oil used. As a consequence, this may result in a reduction in the quality of the oil [23,24]. Typical chemical reactions that take place during deep-fat frying are, e.g., hydrolysis, oxidation, isomerization, and polymerization [25].

Oxidation is one of the basic reactions that fats undergo during storage and their use in technological processes. During frying, the oxidation process is additionally accelerated many times by the action of high temperature [26]. As a consequence of the interaction between oxygen from the air and high temperatures, the formation of degradation products with varying molecular weights is observed. As a result of the breakdown of fats, compounds with a lower molecular weight can be formed, such as volatile compounds (aldehydes, ketones, hydrocarbons, and others) and short-chain triacylglycerol monomers. Oxidation can result in the formation of oxidized triacylglycerols and cyclic monomers. As a result of polymerization, high molecular weight compounds are formed, i.e., dimers and oligomers of triacylglycerols [27]. According to the literature, the best method for assessing the quality of frying fats is the analysis of total polar compounds and polymer triglycerides [6].

With the above information considered, the goal of this research was testing the thermal stability of novel oil blends with a 5:1 ratio of *ω*6/*ω*3 fatty acids, as well as their degradation during heating. The fatty acid compositions were determined by gas chromatography (GC), while the content of polar compounds and polymers of triacylglycerols was analyzed by liquid chromatography (LC). The water content was determined by the Karl Fischer method, and oxidative stability was determined by differential scanning calorimetry (DSC) as well as low-field nuclear magnetic resonance (LF NMR) methods.

## 2. Materials and Methods

### 2.1. Materials

The materials used for the analysis consisted of eight novel oil blends, characterized by a 5:1 ratio of *ω*6/*ω*3 fatty acids. These oil blends were obtained by mixing oils that were cold-pressed (rapeseed oil, black cumin oil, milk thistle seed oil, pumpkin seed oil, black cumin oil, hemp oil, linseed oil) and refined (rice bran oil). The fresh, cold-pressed oils were obtained from SemCo (Śmiłowo, Poland), and rice bran oil was purchased from the local market. After mixing the oils, the new blends were left at room temperature for 12 h and then stored at 5 °C until analysis. The composition of the prepared oil blends is shown in Table 1.

### 2.2. Heating Procedure

Each blend was heated in a thin layer model using a 20 cm diameter steel pan at temperatures of 170 °C and 200 °C (±5 °C) for 10 min. This time was chosen as the maximum frying time to produce the finished product while avoiding negative degradation changes. The oil was preheated to a set temperature for 7 and 9 min using magnetic stirrers (IKA RET basic, MS-H-Pro, IKA Works, Inc. Wilmington, NC, USA) and the temperature was monitored with an electronic thermometer. The heating process was repeated twice. After heating, the oil samples were sealed under nitrogen and frozen at −24 °C until analysis.

### 2.3. Chemical Characteristics of Fresh and Heat-Treated Oil Blends

The fatty acid composition was determined according to the AOCS Official Method Ce 1h-05 [28]. Total polar compounds in the oil were analyzed according to the AOCS Official Method 982.27 [29]. The polymers of triacylglycerol (TAG polymer) composition was determined according to AOCS Official Method 993.25 [30]. The determination of the iodine value was conducted according to the AOCS Official Method Cd 1c-85 [31] and calculated (CIV) from fatty acid composition. The water content in the analyzed oil blends was determined by the Karl Fischer method in accordance with the ISO 12937 method [32], using a 831 KF Coulometer (Metrohm AG, Herisau, Switzerland) with a 703 Ti Stand magnetic stirrer (Metrohm AG, Herisau, Switzerland) and a WAX110 balance (Radwag, Radom, Poland).

### 2.4. Nutritional Quality Indicators of Oils

The ratio of polyunsaturated fatty acids to saturated fatty acids (PUFA/SFA) was calculated as an index, which was used to inform about the impact of diet on cardiovascular health.

The index of atherogenicity (IA) measures the relationship between the total amount of saturated fatty acids (SFA) and unsaturated fatty acids (UFA) in food and it was calculated based on the formula:

$$IA=\frac{C12:0+\left(4\times C14:0\right)+C16:0}{\sum UFA}$$

The index of thrombogenicity (IT) determines the relationship between pro-thrombogenic (SFA) and anti-thrombogenic fatty acids (MUFA, *ω*3, and *ω*6 PUFA), and it was calculated by using the following formula:

$$IT=\frac{C14:0+C16:0+C18:0}{\left(0.5\times \Sigma MUFA)+(0.5\times \Sigma \;\omega 6\;PUFA)+(3\times \Sigma \;\omega 3\;PUFA)+(\omega 3/\omega 6\right)}$$

The hypocholesterolemic/hypercholesterolemic (HH) ratio is the relationship between sum of oleic acid (C18:1) and polyunsaturated fatty acids (hypocholesterolemic part), and saturated fatty acids from C12:0 to C16:0 (hypercholesterolemic part) and it was calculated according to the formula:

$$HH=\frac{cis-C18:1+\Sigma PUFA}{C12:0+C14:0+C16:0}$$

### 2.5. LF NMR Studies

The spin–lattice (T_1_) and spin–spin (T_2_) relaxation times were measured by using pulse NMR spectrometer PS15T (ELLAB, Poznań, Poland) which operates at 15 MHz at a temperature of 20.0 ± 0.5 °C. The inversion-recovery (π − t − (π/2)) pulse sequence [33] was applied to T_1_ relaxation times measurements. Distances (t) between RF pulses were varied in the range of 0.2 to 90 ms at a repetition time of 15 s. For each configuration, a total of 32 FID signals and 119 points for each FID signal were collected. The CPMG spin echoes ((π/2) − t − (π)_n_) were used for determination of the T_2_ relaxation times. The distance t between the RF pulses was 2 ms and the repetition time was 15 s. The number of spin echoes (n) was 100. Mean correlation times (τ_c_) were calculated used the method described by Małyszek et al. [34].

### 2.6. Oxidative Stability of the Blends Measured by DSC

The oxidation induction time of the oil blends was determined by differential scanning calorimetry in a DSC 3 differential scanning calorimeter (Mettler-Toledo GmbH, Greifensee, Switzerland). For this purpose, an oil sample (16 ± 1 mg) was heated to 130 °C at a rate of 10 °C/min in nitrogen flow (50 mL/min) and this temperature was maintained for 5 min under a nitrogen atmosphere. Then, the samples were oxidized in isothermal conditions at 130 °C in an open crucible with a constant flow of oxygen (50 mL/min) until a rapid increase in the measurement signal was obtained. The oxidation induction time (isothermal OIT) was determined by the tangent method using STARe Evaluation 16.3 software (Mettler-Toledo GmbH, Greifensee, Switzerland). The standard deviation between repeated determinations of the same sample did not exceed 10%.

### 2.7. Statistical Analysis

Two independent experiments were performed, and all tests were repeated in duplicate (n = 4). Mean values and standard deviations were calculated with Microsoft Excel 2021 (Microsoft Corporation, Redmond, WA, USA). Statistica 13.3 (Dell Software Inc., Round Rock, TX, USA) and R software (version 4.1 with packages FactoMineR v.2.4 and FactoExtra v.1.0.7) were used for principal components analysis (PCA). To calculate significant differences between means (*p* < 0.05, analysis of variance ANOVA) we used Tukey’s multiple range test.

## 3. Results and Discussion

### 3.1. Composition of Fatty Acids in the Analyzed Blends

Vegetable oils are a rich source of many valuable fatty acids, particularly unsaturated ones. Ensuring the appropriate quantity and, more importantly, the quality of fat consumption is a crucial nutritional challenge. It seems particularly important in plant-based diets, which are often poor in essential *ω*3 acids. Therefore, it is important that the fats consumed have an appropriate ratio of *ω*3 to *ω*6 fatty acids to ensure their nutritional value [35,36,37]. The chromatographic analysis confirmed that the developed oil blends are characterized by a favorable *ω*6/*ω*3 ratio in a range from 4.84 to 5.25 to 1 (Table 2). WHO/FAO proposes an optimal ratio of *ω*6 to *ω*3 of 5:1–10:1 [38]. Therefore, the oil blends obtained and analyzed in this report are in line with the general recommendations. It is worth mentioning, however, that in different countries other recommendations are also used, but in each of them attention is paid to reducing the share of *ω*6 acids and increasing the share of *ω*3 acids [13,14,39].

**Figure 1:**
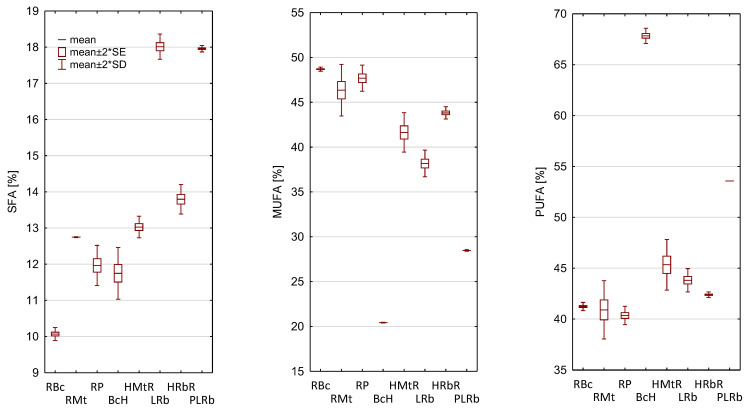
The composition of the main group of fatty acids of the analyzed blends. Table 1 presents the composition of the blends.

The analysis of fatty acid profile indicated that in each of the analyzed blends, two fatty acids were dominant: oleic acid (C18:1, *ω*9) and linoleic acid (LA, C18:2, *ω*6). However, ALA accounted for the third largest share of fatty acid, with the largest amount, as much as 11.43%, contained the BcH blend (Table 2). This blend also contained the most PUFA and the least MUFA (Figure 1). Including oils rich in ALA in the diet can significantly reduce the deficits in the consumption of *ω*3 fatty acids in modern diets, however, it is important to ensure adequate stability of the oils due to the susceptibility of ALA to oxidation. The calculating iodine number (CIV) can be calculated, based on the amount of unsaturated fatty acids in oils, to determine the oil stability (Table 2). The more unsaturated fatty acids, the higher the CIV value and the greater the susceptibility of the oil to oxidation [40]. Due to the highest content of PUFA, the BcH blend is characterized by the highest CIV value, which proves that among all the analyzed blends it is the most susceptible to oxidation. The highest SFA content and the lowest CIV value were recorded for LRb. It can therefore be assumed that this blend will be the most stable during heat treatment [41].

### 3.2. Nutritional Quality Indicators of Oils

The PUFA/SFA index is used to evaluate the effect of diet on cardiovascular health. Increasing the daily intake of PUFAs can decrease the levels of low-density lipoprotein cholesterol and serum cholesterol, while increasing the intake of SFAs can raise serum cholesterol levels. A higher PUFA/SFA ratio is considered to have a more positive impact on cardiovascular health [42]. The highest value of 5.77 was recorded for BcH and the lowest was recorded for LRb (2.43) (Table 3). Nevertheless, each of the analyzed oil blends was characterized by very high values of this coefficient. For comparison, the values for meat range from 0.11 to 2.04, for fish from 0.50 to 1.62, and for shellfish from 0.20 to 2.10 [42]. Ulbritcht and Southgate [43] proposed a new index called IA to assess the atherogenicity of foods because the PUFA/SFA ratio was too general and unsuitable. They based the IA index on the PUFA/SFA ratio and available evidence and then verified if the resulting values were consistent. Unsaturated fatty acids (UFAs) can help prevent the buildup of plaque and lower levels of phospholipids, cholesterol, and esterified fatty acids, making them anti-atherogenic. Eating foods or products with a lower IA index can decrease the total cholesterol and LDL-C levels in human blood plasma [44,45]. All the analyzed oil blends were characterized by low IA values (Table 3), typical for oils, ranging from 0.07 to 0.19, which is consistent with the literature data for vegetable oils. Filip et al. analyzed the quality of sunflower oil, showing that the IA for this oil was 0.09–0.11 [46]. In addition, Ulbritcht and Southgate [43] also proposed an index of thrombogenicity, which describes the relationship between pro-thrombogenic fatty acids and anti-thrombogenic fatty acids, indicating that the lower the IT of a food, the more beneficial it is for our health. As expected, this indicator also clearly indicates the pro-health properties of the analyzed oils, which have properties to prevent cardiovascular diseases. The HH ratio shows the connection between fatty acids that lower cholesterol and those that raise it, which can be found in oils and foods. The cholesterol-lowering part includes PUFA and oleic acid, while the cholesterol-raising part includes saturated acids from C12:0 to C16:0 [42]. The HH ratio of the analyzed oil blends ranged from 5.20 to 13.01. It can therefore be concluded that five (RBc, RMt, RP, BcH, and HMtR) out of the eight analyzed blends have a higher nutritional value, and their consumption can effectively help prevent cardiovascular diseases. However, it is worth clearly pointing out that the discussed indicators are used only for a general assessment of the potential nutritional value of fats and do not clearly indicate the pro-health properties of the oil. After the initial assessment of oils using the discussed indicators, more thorough research is needed to determine the effects of oils on health.

### 3.3. Influence of the Heating Process on the Formation of Total Polar Compounds (TPC) and Polymerized Triacylglycerols (PTG)

When oils are heated, the amount of TPC in them increases. The lower the TCP content, the more stable the oil is when heated [25]. Changes in the TPC content in unheated and heated oil blends are presented in Table 4. The lowest content of TPC in oil blends before the heating process was demonstrated for LRb (1.84%) and the highest content was demonstrated for RP (6.45%). A significant increase in TPC was observed during heating of all the analyzed blends. This increase was more intense when using higher heating temperatures. Surprisingly, the LRb blend, which was characterized by the lowest TPC content in the initial stage (unheated), showed the lowest TPC content only after heating at 170 °C. In the case of using a higher temperature (200 °C), the HRbR sample (7.88%) exhibited a much higher TPC content in the unheated blend than in LRb the sample (2.54 vs. 1.84% for HRbR and LRb, respectively). The highest content of TPC during heating at 170 °C was found in RP (10.54%), and in the temperature of 200 °C HMtR (14.98%) and RP (14.53%). An increase in the concentration of TPC in heated oils is a common phenomenon and often occurs when food is fried. The high temperature used during the frying process, but also the water from the fried raw materials or oxygen from the air, favor the formation of polar components [25,47]. Importantly, none of the analyzed blends exceeded the permissible TPC content limits, which in many countries were set at 24–25% [6]. The observed increase in the concentration of TPC after the heating process results from the changes that fatty acids undergo through hydrolysis, oxidation, or polymerization [48]. It is worth noting, however, that not only the profile of fatty acids affects the formation of TPC, but the protective substances present in oils (antioxidants such as tocochromanols or polyphenols) can significantly reduce the rate of TPC formation during frying or heating the oil [49].

**Figure 2:**
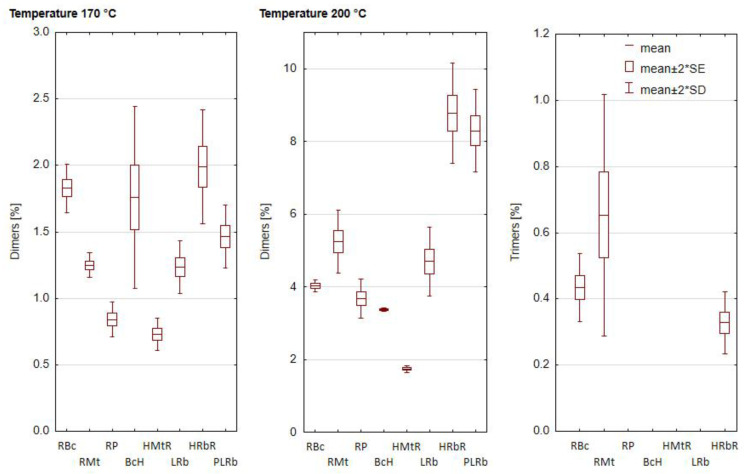
Composition of dimers and trimers of triacylglycerols (TAG) of oil blends heated at 170 and 200 °C. SD—standard deviation, SE—standard error. Table 1 presents the composition of the blends.

The presence of PTG was not observed in the analyzed fresh, unheated blends of oils (Figure 2). However, the polymerization of triacylglycerols occurs when oils are heated, and as a result of this reaction, which is radical in nature, compounds with a molecular weight of 690 to even 1600 Daltons are formed [50]. TAG dimers were observed in all samples at both temperatures. Their content depended on the type of blends and the heating temperature. The factor significantly differentiating the polymer content was temperature. During heating at 170 °C, the TAG dimers content increased from 0.73 to 1.99%. The lowest dimer content was in HMtR, and the highest in HRbR. Heating at 200 °C led to a sharp increase in TAG dimers. Their content ranged from 1.74 to 8.77% and depended on the type of blends. Depending on the sample heated, an increase in heating temperature led to an increase in the content of dimers from 2 to 7 times. TAG trimers were present only in three samples heated at 200 °C. TAG trimer content was 0.33, 0.44, and 0.65% in HRbR, RBc and RMt, respectively. The high content of PUFA in oil can significantly increase the rate of reaction in the PTG formation process [51]. Surprisingly, however, the oil blend with the lowest dimer content (HMtR) after heating both at 170 °C and 200 °C did not contain the lowest content of PUFA. As Lampi and Kamal-Eldin [52] indicate, the formation of PTG, apart from the PUFA content, is also significantly influenced by antioxidant compounds, both polyphenolic and tocochromanols, which effectively inhibit the thermal degradation of fat and reduce the formation of PTG. It can therefore be assumed that this blend is rich in antioxidant compounds, hence the low content of PTG in the analyzed oil after heating.

### 3.4. LF NMR Relaxometry and DSC Oxidation Induction Time

Water can affect the rate of lipid oxidation and must be monitored. It can cause triacylglycerols to break down into mono- and diacylglycerols and free fatty acids, which can change the structure of water-in-oil microemulsions and act as prooxidants [53]. The development of these specific structures in cold-pressed oils is determined by the balance of minor components such as phospholipids, tocopherols, sterols, and polyphenols. Trace amounts of water were found in the tested blends. The lowest content was recorded for LRb (176.4 ppm), and the highest, amounting to 823.5 ppm, for RP (Table 5). However, even if there are high levels of water (up to 1000 ppm), oxidation may not increase if the water bonds with polar compounds and gets trapped in multilayer association colloids [53].

Relaxation time values are typical for oils, however, the differences observed in the tested samples (Table 5) result from the different content of individual fatty acids and the presence of water molecules. The fact that one T_1_ relaxation time and one T_2_ relaxation time is observed means that, from a molecular point of view, no distinction is made between the movements of the fatty acid proton fraction and the water proton fraction. This is due to the hydrophobic-hydrophilic interactions of fatty acid chains and water molecules [54].

**Figure 3:**
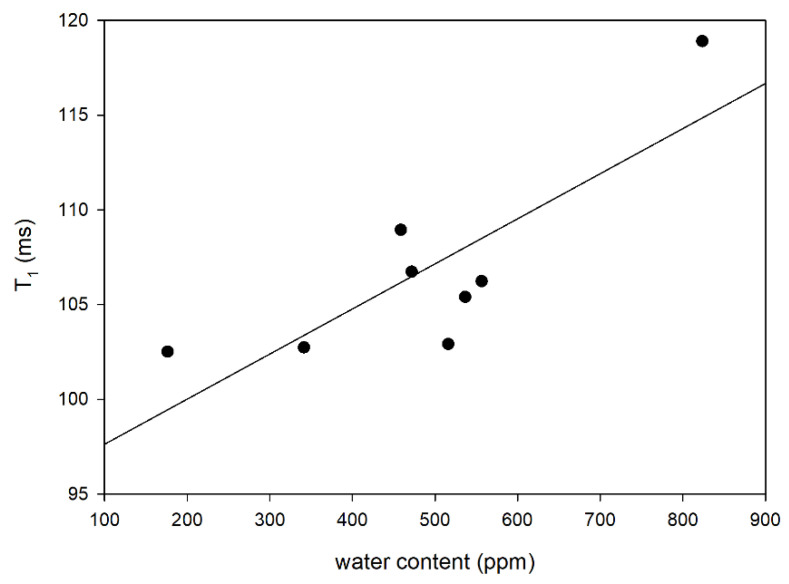
The dependence between water content and spin-lattice relaxation time (T_1_) in the analyzed blends (R^2^ = 0.7825).

It was found that the amount of water determined in the system is correlated with the values of spin-lattice relaxation times. With the increase in the amount of water determined by the Karl Fischer method, greater values of this time are recorded (Figure 3). The lowest water content was found in this blend, which is characterized by the most flexibly fatty acid chains. Thus, the obtained results suggest that the molecules form lamellar structures with the acid chains, which leads to their stiffening. The water content in the blends directly proportionally extends the T_1_ time, which describes the return of the excited proton system to the equilibrium state. The presence of water molecules that do not interact with other protons in the system has the greatest influence on the T_1_ values [55,56]. The observed linear relationship primarily confirms the presence of water in the blends.

**Figure 4:**
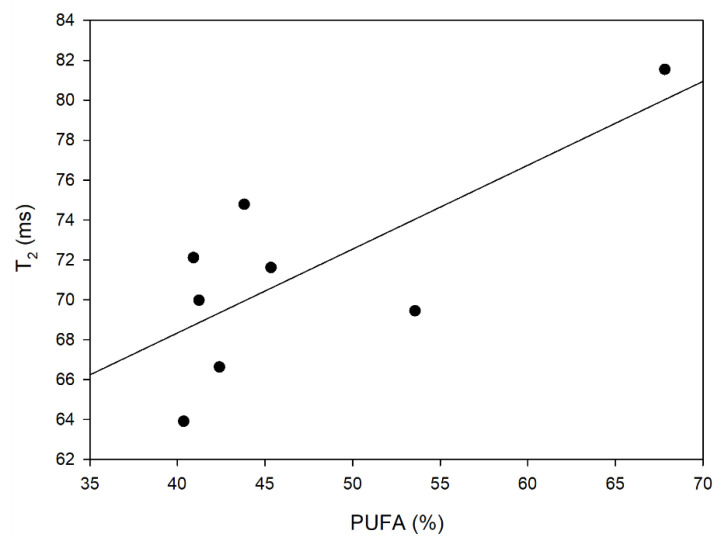
The dependence between polyunsaturated fatty acid (PUFA) contents and spin-spin relaxation time (T_2_) in the analyzed blends (R^2^ = 0.7039).

However, the presence of water particles in the blend cannot be unequivocally linked to the proton movements of fatty acid chains. However, a relationship was found between the content of polyunsaturated fatty acids and the molecular dynamics of protons (Figure 4), which means that the spin-spin relaxation time increases with the increase in the amount of PUFA.

Based on the spin-lattice and spin-spin relaxation times, the mean correlation times (τ_c_) were calculated (Table 5). These are microscopic parameters reflecting the possibility of the rotational motion of molecules containing protons. For pure water, a system in which protons have the greatest possibility of movement, this parameter takes values of the order of 10^−12^ s. In ice, an ordered structure with a significantly limited possibility of proton rotation, mean correlation time values of 10^−6^ s are noted [57]. The smallest value of τ_c_ was noted for the LRb sample. It can therefore be concluded that this blend is characterized by high mobility of fatty acid chains. Ordered fatty acid chains with the smallest proton molecular dynamics are found in the RP blend.

Based on the GC analysis results presented in Table 2 (see Section 3.1), it can be seen that the analyzed oils are rich in essential fatty acids. Oxidation (self-oxidation and during heat treatment) of polyunsaturated fatty acids is mainly responsible for changes in the quality of oils during storage and use. There are many methods for analyzing oxidative changes in oils, including Rancimat, peroxide value (PV), anisidine value (AV), and spectrophotometric analyses, however, due to the fact that oil oxidation is an exothermic reaction, these changes can also be monitored by differential scanning calorimetry [58,59]. As mentioned, the oxidation induction time (OIT) of the analyzed blends was analyzed, and the results of the isothermal determination of the oxidative stability of oils by DSC analysis were presented in Table 5. It can be observed that RP possessed the longest OIT, with the shortest being recorded for BcH. The longer the OIT of the analyzed oil, the higher its oxidative stability [60,61].

**Figure 5:**
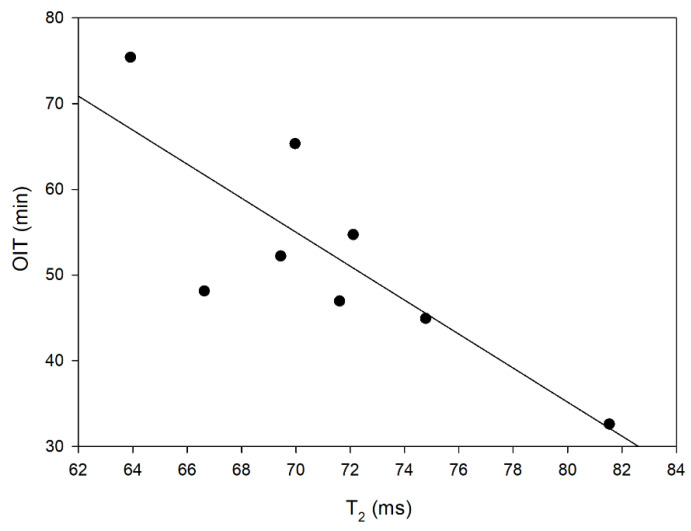
The dependence between spin-spin relaxation time (T_2_) and oxidation induction time (OIT) in the analyzed blends (R^2^ = 0.7901).

The observed changes in the oxidation time of the blends, measured by the DSC method, can be related to the results obtained by the LF NMR method. It has been shown that with the increase in the freedom of rotation of protons in the analyzed blends (described with the time T_2_), the oil oxidation time decreases (Figure 5). Such a relation may suggest that blends in which fat protons have a large freedom of movement and which contain a significant amount of water molecules, the water does not form lamellar structures [62], so the measured oxidation time decreases, which directly translates into lower durability of the oil. Both methods of analysis, DSC and LF NMR, are based on the molecular properties of matter. The finding of an inversely proportional relationship between OIT and T_2_ means that OIT is determined by the molecular structure of the oil blend [55,56,63]. The freedom of molecular movement of ^1^H contributes to the acceleration of the oxidation process; therefore, the lower the molecular dynamics, the more stable the oil.

**Figure 6:**
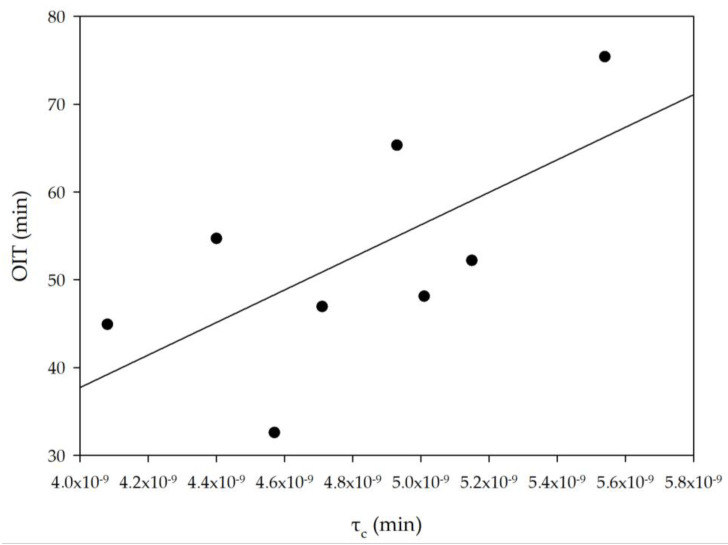
The dependence between oxidation induction time (OIT) contents and the mean correlation time in analyzed blends (R^2^ = 0.6859).

In addition, the increase in mean correlation times is directly related to the ordering of the structure, which directly translates into an increase in the value of OIT (Figure 6). It can therefore be concluded that the stability of blends should be associated with molecular parameters describing the dynamics of proton movements, which in turn is directly related to the presence of bulk water molecules, a fraction whose molecules do not interact directly with the protons of fatty acids.

### 3.5. Statistical Considerations on the Influence of the Analyzed Parameters on the Stability of Oils

**Figure 7:**
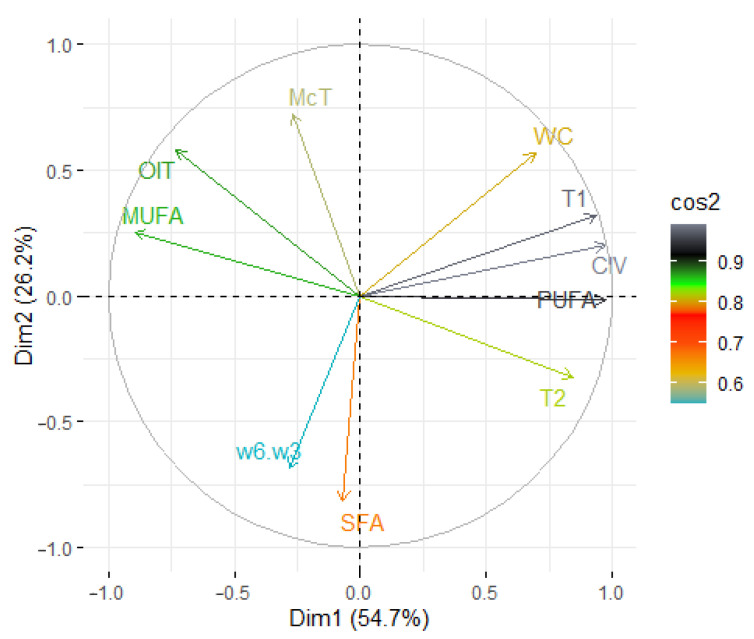

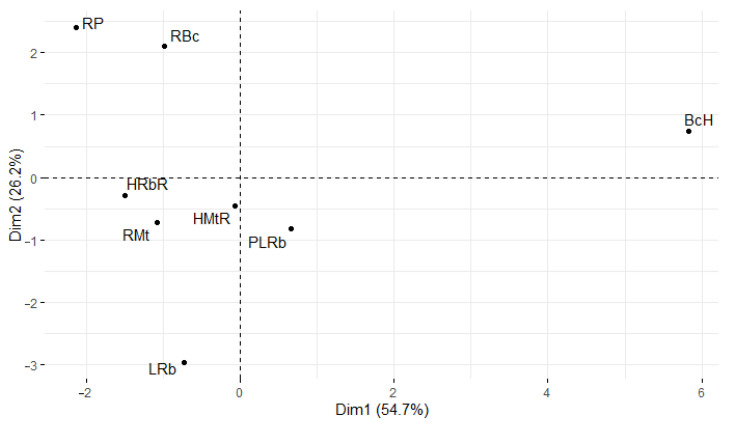
Principal component analysis (PCA) of the loadings plot and the score plot of data from saturated fatty acids (SFA), monounsaturated fatty acid (MUFA), polyunsaturated fatty acid (PUFA), calculated iodine value (CIV), *ω*6/*ω*3 fatty acid ratio, water content, T_1_ and T_2_ relaxation time, and oxidation induction time (OIT) in the unheated oil blends.

The principal component analysis (PCA) was applied to observe possible clusters in the fresh and heated oils blends at 170 °C and 200 °C. The PCA of fresh, unheated, samples show that the first two dimensions accounted for 80.9% (Dim1 = 54.7 and Dim2 = 26.2%) (Figure 7). Factor 1 is mainly correlated with T_1_ relaxation time (r = 0.936), PUFA (r = 0.975), and CIV (r = 0.976), and negatively correlated with MUFA share (r = −0.896). Factor 2 was negatively correlated with SFA share (r = −0.818). In the score plot we can observe data divided into four groups. The biggest group is located in the center of score plots and include four samples (HRbR, HMtR, PLRb, and LRb). The more interesting groups are located on the outskirts of the plot. The most distant group is the BcH sample. It is located in the right of the Y axis. This sample was characterized by the highest water content (823.5 ppm), the shortest oxidation induction time (32.6 min), the longest T_1_ (118.9 ms) and T_2_ (81.54 ms) relaxation times, the lowest share of MUFA (20.42%), and the highest share of PUFA (67.83%) compared to the other samples. The last two groups are located on the left of the Y axis. One, which includes the RP and RBc samples, is located high above the X axis. These two samples were characterized by a long oxidation induction time (75.4 and 65.33 min, respectively), the highest share of MUFA (47.68% and 48.70%, respectively), and the lowest share of PUFA (40.35% and 41.23%, respectively). The four groups are located under the X axis and included the LRb samples with the lowest water content (176.4 ppm), and a comparatively short oxidation induction time (44.93 min).

**Figure 8:**
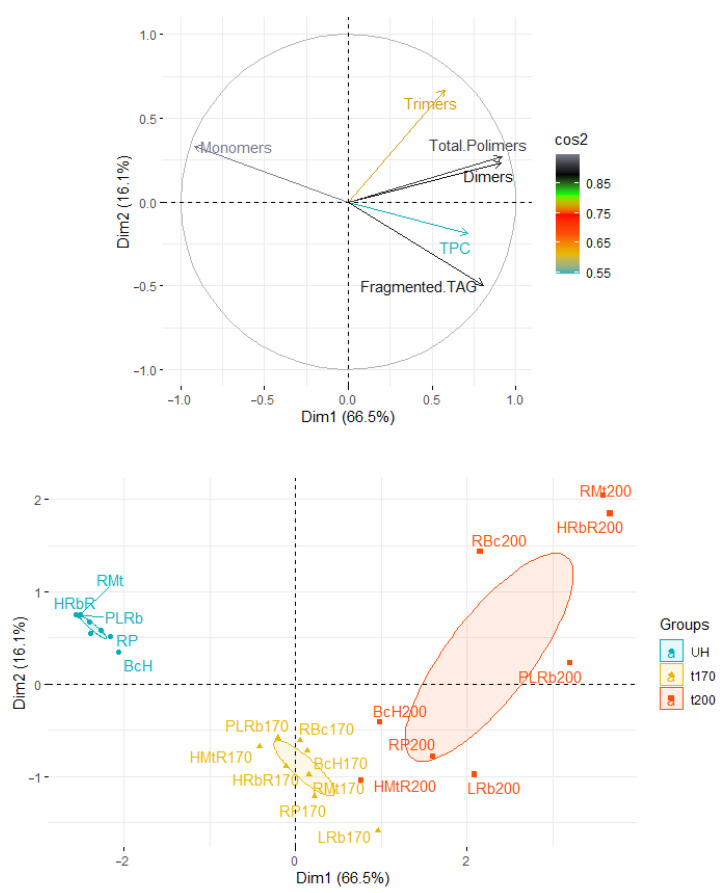
Principal component analysis (PCA) of the loadings plot and the score plot of data from total polar compounds (TPC) content and polymerization of triacyclglycerols composition in unheated oil blends and heated at 170 °C and 200 °C. UH—not heated blends, t170 and t200 oils blends heated at 170°C and 200 °C.

The PCA analysis of the heated oils samples show that the two first dimensions accounted for 82.6% (Dim1 = 66.5 and Dim2 = 16.1%). The distribution of the oil samples depending on the heating temperature is shown in Figure 8. Factor 1 is mainly correlated with dimers (r = 0.911) and total polymers content (r = 0.916) and negatively correlated with monomers content (r = −0.915). In the score plot we can observe three groups of samples. The unheated blends are located on the left side of the Y. These samples are characterized by a low level of TPC (from 1.84 to 6.45%) and no presence of TAG polymers. The following two groups included samples after the heating process. However, when the temperature was 170 °C, the oil samples were located under the X axis and the dispersion of samples was small. Samples heated at 200 °C are characterized by greater variability. We can observe four samples close to the samples heated in a lower temperature (BcH, RP, HMtR, and LRb) and four extremely distant samples (RMt, HRbR, RBc, and PLRb). Their large distance from the other heated samples results from the presence of TAG trimers (RMt, HRbR and RBc) and very high content of TAG dimers (PLRb).

## 4. Conclusions

One way to reduce the consumption of animal fats with high levels of saturated fatty acids is to include more vegetable oils in our diet. These oils can provide a significant amount of essential fatty acids. Unfortunately, the higher the PUFA content, the lower the stability of the oils during storage and heat treatment. As part of this research, eight new compositions of oil blends were developed, which were characterized in terms of changes during heating. It has been shown that from a nutritional point of view, these blends are characterized by a favorable ratio of *ω*6/*ω*3 fatty acids, which may potentially have a beneficial effect on the prevention of cardiovascular diseases. In addition, it was shown that these blends are characterized by high thermal stability, even when heated at high temperatures. Although heating resulted in an increase in TPC and TAG compared to fresh oils, the observed levels of the analyzed compounds do not exceed the acceptable standards for oils for consumption. The high stability of the blends was also confirmed by DSC and LF NMR methods. Since there are many compounds in the studied oil blends, such as phenolic acids, sterols, and tocochromanols, which are suggested to contribute to the protection of oils, further research on their activity could improve our knowledge about the mechanisms underlying the beneficial effects observed in the current experiments.

## Figures and Tables

**Table 1:** Oil blend compositions.

Code	Type of Oil			Percentage (%)		
RBc	Rapeseed oil	Black cumin oil	-	60	40	-
RMt	Rapeseed oil	Milk thistle seed oil	-	50	50	-
RP	Rapeseed oil	Pumpkin seed oil	-	58	42	-
BcH	Black cumin oil	Hemp oil	-	40	60	-
HMtR	Hemp oil	Milk thistle seed oil	Rapeseed oil	10	50	40
LRb	Linseed oil	Rice bran oil	-	11	89	-
HRbR	Hemp oil	Rice bran oil	Rapeseed oil	15	55	30
PLRb	Pumpkin seed oil	Linseed oil	Rice bran oil	60	15	25

**Table 2:** Fatty acid composition (%) and calculated iodine value (CIV) of oil blends.

Oil Blends ^1^	C16:0	C16:1	C18:0	C18:1	C18:2	C18:3	C20:0	C20:1	C22:0	Ratio *ω*6/*ω*3	CIV
RBc	7.76 ± 0.13	0.18 ± 0.03	2.21 ± 0.04	48.52 ± 0.08	34.17 ± 0.26	7.07 ± 0.06	0.10 ± 0.01	nd	nd	4.84:1	119.56 ± 0.20
RMt	6.52 ± 0.05	0.12 ± 0.01	3.88 ± 0.05	46.23 ± 1.45	34.36 ± 1.09	6.55 ± 0.34	1.55 ± 0.07	nd	0.80 ± 0.01	5.25:1	116.51 ± 1.55
RP	8.23 ± 0.24	0.15 ± 0.01	3.57 ± 0.04	47.54 ± 0.73	33.65 ± 0.46	6.70 ± 0.01	0.14 ± 0.01	nd	0.03 ± 0.01	5.02:1	116.83 ± 0.14
BcH	8.56 ± 0.34	0.12 ± 0.03	2.69 ± 0.04	18.46 ± 0.08	56.40 ± 0.54	11.43 ± 0.16	0.37 ± 0.01	1.84 ± 0.07	0.13 ± 0.02	4.93:1	145.03 ± 0.49
HMtR	6.65 ± 0.24	0.11 ± 0.00	3.96 ± 0.07	41.22 ± 1.10	38.06 ± 0.87	7.27 ± 0.38	1.60 ± 0.13	0.31 ± 0.00	0.82 ± 0.03	5.24:1	120.75 ± 1.55
LRb	15.64 ± 0.06	0.13 ± 0.03	1.79 ± 0.12	37.49 ± 0.81	36.67 ± 0.95	7.14 ± 0.38	0.57 ± 0.00	0.55 ± 0.03	0.01 ± 0.00	5.14:1	115.00 ± 0.02
HRbR	11.61 ± 0.09	0.14 ± 0.02	1.75 ± 0.08	42.88 ± 0.33	35.47 ± 0.02	6.92 ± 0.16	0.41 ± 0.03	0.80 ± 0.00	0.03 ± 0.00	5.13:1	117.17 ± 0.08
PLRb	12.91 ± 0.07	0.11 ± 0.00	4.63 ± 0.10	28.21 ± 0.05	44.93 ± 0.05	8.64 ± 0.05	0.37 ± 0.01	0.16 ± 0.01	0.05 ± 0.00	5.20:1	124.90 ± 0.09

^1^ Table 1 presents the composition of the blends. Values are means of four determinations ± SD. nd—not detected.

**Table 3:** Nutritional index values used to assess the nutritional quality of the oil blends.

Oil Blends ^1^	PUFA/SFA	IA	IT	HH
RBc	4.09	0.09	0.15	11.57
RMt	3.21	0.07	0.16	13.36
RP	3.37	0.09	0.18	10.68
BcH	5.77	0.10	0.14	10.08
HMtR	3.48	0.08	0.16	13.01
LRb	2.43	0.19	0.27	5.20
HRbR	3.07	0.13	0.20	7.34
PLRb	2.98	0.16	0.26	6.33

^1^ Table 1 presents the composition of the blends. The indices were calculated using the average values of fatty acid composition of analyzed blends.

**Table 4:** Total polar content (TPC) in unheated and heated oil blends (%).

Oil Blends ^1^	Unheated	Heating Temperature (°C)	
		170	200
RBc	5.41 ± 0.10 ^aE^	7.41 ± 0.14 ^bCD^	10.74 ± 0.17 ^cB^
RMt	3.78 ± 0.14 ^aC^	8.99 ± 0.10 ^bE^	11.74 ± 0.14 ^cC^
RP	6.45 ± 0.30 ^aF^	10.54 ± 0.13 ^bG^	14.53 ± 0.23 ^cF^
BcH	4.98 ± 0.15 ^aDE^	7.84 ± 0.17 ^bD^	12.74 ± 0.06 ^cD^
HMtR	4.62 ± 0.16 ^aD^	9.57 ± 0.17 ^bF^	14.98 ± 0.27 ^cF^
LRb	1.84 ± 0.14 ^aA^	4.01 ± 0.11 ^bA^	7.96 ± 0.04 ^cA^
HRbR	2.54 ± 0.04 ^aB^	5.29 ± 0.13 ^bB^	7.88 ± 0.23 ^cA^
PLRb	3.41 ± 0.04 ^aC^	7.05 ± 0.14 ^bC^	13.45 ± 0.00 ^cE^

^1^ Table 1 presents the composition of the blends. Values are means of four determinations ± SD. Means in the same row, followed by different small letters indicate significant differences (*p* < 0.05) between the same samples with different heating temperatures. Means in the same column, followed by different capital letters, indicate significant differences (*p* < 0.05) between samples in the same heating temperature.

**Table 5:** Results of the water content, LF NMR relaxometry, and DSC analyses of oil blends.

Oil Blends ^1^	Water Content (ppm)	T_1_ Relaxation Time (ms)	T_2_ Relaxation Time (ms)	τ_c_ (s)	OIT (min)
RBc	536.5 ± 4.9 ^b^	106.73 ± 0.33 ^c^	69.97 ± 1.22 ^d^	4.9298 × 10^−9^	65.33
RMt	471.7 ± 6.6 ^c^	102.91 ± 0.43 ^d^	72.11 ± 1.56 ^cd^	4.4037 × 10^−9^	54.72
RP	823.5 ± 10.5 ^a^	105.40 ± 0.38 ^c^	63.91 ± 1.40 ^f^	5.5434 × 10^−9^	75.40
BcH	516.1 ± 18.1 ^b^	118.90 ± 0.33 ^a^	81.54 ± 1.42 ^a^	4.5746 × 10^−9^	32.60
HMtR	341.4 ± 14.6 ^d^	106.23 ± 0.34 ^c^	71.61 ± 1.41 ^d^	4.7103 × 10^−9^	46.95
LRb	176.4 ± 4.7 ^e^	102.51 ± 0.28 ^d^	74.78 ± 1.45 ^bc^	4.0809 × 10^−9^	44.93
HRbR	556.6 ± 20.9 ^b^	102.74 ± 0.29 ^d^	66.63 ± 1.23 ^e^	5.0148 × 10^−9^	48.13
PLRb	458.6 ± 13.6 ^c^	108.95 ± 0.32 ^b^	69.44 ± 1.34 ^d^	5.1513 × 10^−9^	52.20

^1^ Table 1 presents the composition of the blends. Values are means of four determinations ± SD. Means in the same column, followed by different small letters indicate significant differences (*p* < 0.05) between the same samples. τ_c_—mean correlation time, OIT—oxidation induction time.

## Data Availability

The datasets generated for this study are available on request to the corresponding author.
